# Proteinuria and proximal tubular epithelial cells: correlation between immunofluorescence, histology, and degree of proteinuria

**DOI:** 10.3389/fneph.2024.1469388

**Published:** 2024-10-31

**Authors:** Maria Bernadette CY Chow, Vedat Yildiz, Laura Biederman, Alana Dasgupta, Anjali A. Satoskar, Aaron Chow, Tibor Nadasdy, Sergey V. Brodsky

**Affiliations:** ^1^ Department of Pathology, The Ohio State University Wexner Medical Center, Columbus, OH, United States; ^2^ Deparment of Pathology, North District Hospital, Hong Kong, Hong Kong SAR, China; ^3^ Center for Biostatistics, The Ohio State University Wexner Medical Center, Columbus, OH, United States; ^4^ Department of Pathology, Nationwide Children Hospital, Columbus, OH, United States

**Keywords:** proteinuria, renal pathology, immunofluorescence, proximal tubular epithelial cells, resorption droplets

## Abstract

Proteins are filtered from the blood through the glomerular filtration barrier. Filtered proteins are reabsorbed by proximal tubular epithelial cells (PTECs), which have been shown to possess the ability to regulate protein reabsorption. Histologically, these reabsorbed proteins are seen as tubular protein reabsorption droplets (TPRDs). Experimental studies indicate that PTECs play an important role in regulating proteinuria but the correlations between TPRD and the degree of proteinuria in human kidney biopsies have not been investigated in detail. Consecutive native kidney biopsies with non-proliferative glomerular disease performed at the OSUWMC for a 1-year period were analyzed. Cases with acute glomerular diseases and inadequate biopsies were excluded. The staining intensity and the percentage of TPRDs, as well as other morphologic parameters, were assessed. A total of 109 kidney biopsies were included in the study. A reverse correlation was identified between the percentage of albumin TPRDs and proteinuria (*p* = 0.047). There were positive correlations between proteinuria and the staining intensity for IgG TPRDs (*p* = 0.05) and the degree of acute tubular necrosis (ATN) (*p* = 0.015). In patients with no ATN, positive correlations between proteinuria and albumin and IgG TPRDs were seen, whereas in patients with ATN, these correlations were lost. A positive correlation was seen between proteinuria and chronic kidney injury. A strong correlation was noted between the degree of proteinuria and podocyte foot process effacement. Our data indicate that PTECs regulate proteinuria by absorbing proteins from the urine filtrate. Therefore, based on the human renal biopsy material, our study confirms that well-functioning renal PTECs play an important role in the regulation of proteinuria.

## Introduction

Proteinuria is a known risk factor for developing chronic kidney disease (CKD) (1). The mechanisms by which the proteins are filtered and handled in the kidney have been extensively studied, mostly in animal models. The glomerular filtration barrier (GFB) determines the amount and type of plasma proteins being filtered into the glomerular filtrate. The GFB consists of three major components: the fenestrated endothelium, the glomerular basement membrane (GBM), and the podocytes that have slit diaphragms in between the interdigitating foot processes (2). Under physiological conditions, most of the filtered proteins are reabsorbed within the first two segments of the proximal tubule by proximal tubular epithelial cells (PTECs) mainly via endocytosis (3).

Proteinuria occurs when the amount of filtered proteins exceeds the reabsorption capacity of the PTECs or when there is a dysfunction of the PTECs. Accordingly, it can be classified into glomerular proteinuria and tubular proteinuria. Glomerular proteinuria is further divided into selective proteinuria and non-selective proteinuria. In selective proteinuria, the GFB allows only the passage of low molecular weight proteins (LMWPs), such as albumin, but not high molecular weight proteins (HMWPs), such as immunoglobulins (Ig) (4, 5). On the contrary, in non-selective proteinuria, both LMWPs and HMWPs pass through the GBM into the ultimate glomerular filtrate (5, 6).

Based on an albumin overload animal model, Molitoris et al. demonstrated that reabsorption in the proximal tubule was the major determinant of urinary albumin excretion, rather than alteration in the glomerular filtration of albumin (7). Furthermore, it was shown that the diphtheria toxin-mediated increase in glomerular permeability to albumin resulted in corresponding increases in the albumin reabsorption in the first two segments of the proximal tubules, indicating that PTECs have the capacity to regulate tubular albumin reabsorption and thus the level of urinary albumin (7).

Excessive amounts of proteins in the glomerular filtrate in pathological proteinuria are toxic to tubular epithelial cells (8). Morphologically, the proteins reabsorbed by PTECs appear as tubular protein reabsorption droplets (TPRDs). We hypothesize that there is a correlation between the level of proteinuria and the protein reabsorption droplets in PTECs and the tubular epithelial cell injury. The aim of this study was to investigate the correlation between proteinuria and histologic markers based on human kidney biopsies.

## Materials and methods

Consecutive native kidney biopsies at the Renal and Transplant Pathology Laboratory at the Ohio State University Wexner Medical Center (OSUWMC) that were received from 1 October 2022 to 30 November 2023 were evaluated. Cases with active inflammatory glomerular diseases, such as endocapillary proliferative glomerulonephritis (such as ISN/RPS lupus nephritis class III or IV) or crescentic glomerulonephritis, biopsies with active interstitial inflammation, and biopsies with inadequate sampling or clinical history, were excluded. The cases with active inflammatory glomerular diseases were excluded to avoid the impact of inflammatory cytokines and oxidative stress on the tubular epithelial cells. Each case represented an individual patient. If a patient had several kidney biopsies, then only the first one was used for analysis.

**Figure 1:**
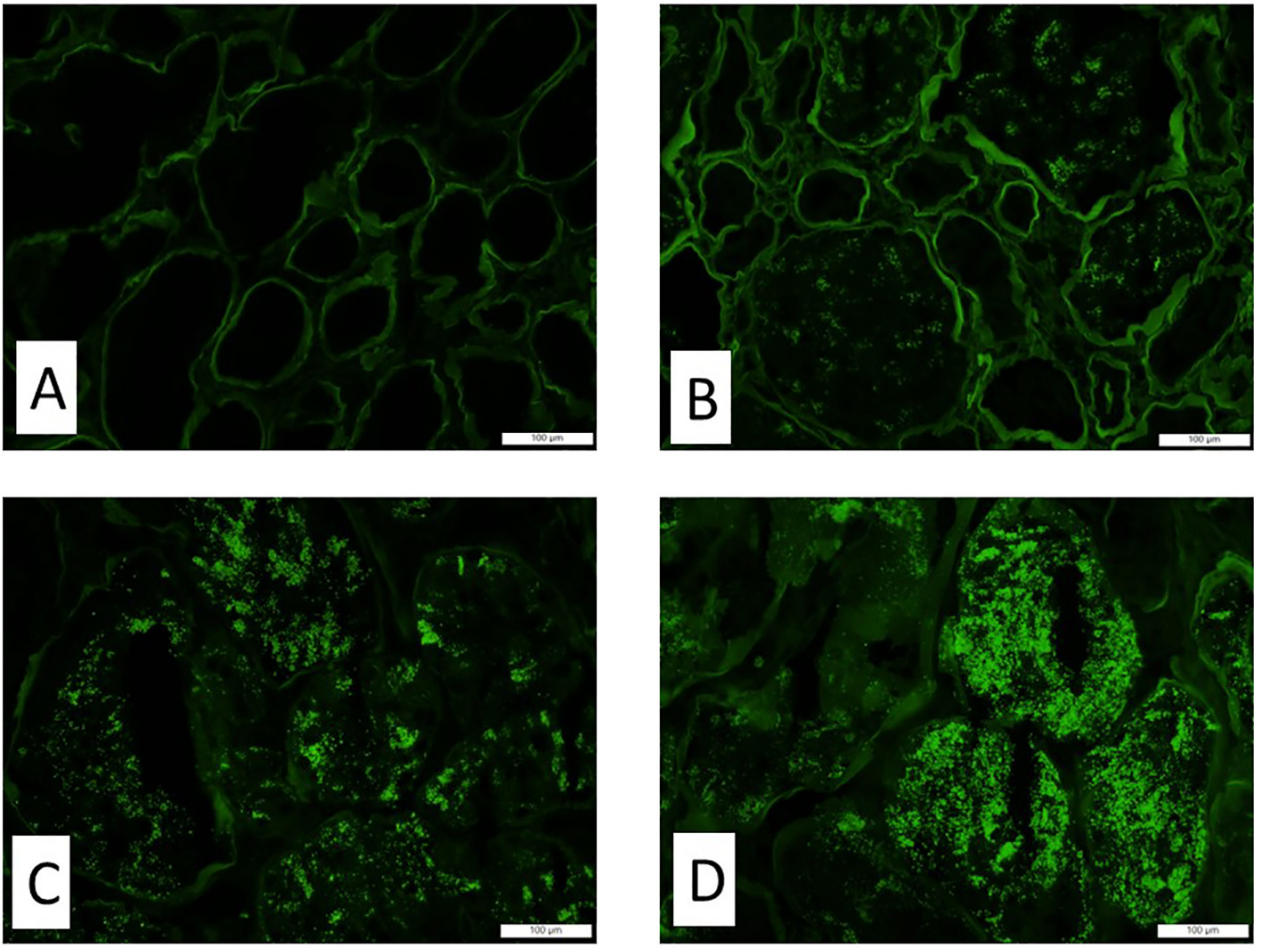
The staining intensity of the tubular protein resorption droplets by immunofluorescence. Representative images of immunofluorescence with antibody and albumin show different staining intensities in tubular protein resorption droplets. **(A)** Score 0, **(B)** score 1, **(C)** score 2, and **(D)** score 3. Magnification ×200.

Each kidney biopsy was routinely processed. Three-micrometer-thick paraffin sections were stained with hematoxylin and eosin (H&E), periodic acid-Schiff (PAS), Masson’s trichrome, and Jones’ Methenamine Silver stain. The following histologic parameters were scored: the total number of glomeruli, the percentage of globally sclerosed glomeruli, the percentage of interstitial fibrosis and tubular atrophy (IFTA), the degree of arterial intimal thickening, the degree of arteriolar hyalinosis, and the degree of acute tubular necrosis (ATN). A semiquantitative scale of 0 to 3 was used to quantify the percentage of the lesions (0 = <10%, 1 = 11%–25%, 2 = 26%–50%, 3 = >50%). Routine direct immunofluorescence was performed on the frozen sections. The staining intensity of TPRDs (with a scale of 0 to 3, as illustrated in **Figure 1**) as well as the percentage of proximal tubules containing these reabsorption droplets was assessed for albumin and IgG. Transmission electron microscopy was performed to assess the extent of podocyte foot process effacement. Demographic data and the levels of proteinuria were collected from clinical records. Urinary protein/creatinine ratio or 24-h proteinuria that was collected within 1 week prior to the kidney biopsy procedure was used.

### Statistical analyses

The data were analyzed using Statistical Analysis Software, version 9.4 (SAS Institute Inc., Cary, NC, USA). The normality of data was assessed after visual inspection of the data and with the Shapiro–Wilk normality test. Continuous data were reported as the median with the interquartile range due to the non-normal distribution of data. Data were analyzed by the Mann–Whitney *U* test for comparison between the proteinuria <3-g and proteinuria ≥3-g groups. Spearman correlation analysis was used for parametric variables to identify the correlation between variables. Results were considered statistically significant if *p*-values were <0.05. The chi-square test was used to analyze differences between diagnostic categories; locally weighted regression analysis was used to fit a smooth curve through points in the scatter plots.

## Results

### Demographic and clinical data

A total of 109 cases were recruited into the study. The major diagnostic categories of the recruited cases are listed in **Table 1**, and the cases were divided into two groups based on the level of proteinuria: group 1 included patients with proteinuria <3 g (47 patients) and group 2 included those with proteinuria ≥3 g (62 patients). It appears that non-immune complex-mediated glomerular diseases were more prevalent in patients with nephrotic range proteinuria, whereas other diseases were more commonly seen in patients with subnephrotic proteinuria (**Table 1**, chi-square = 19.1, *p* < 0.001).

**Table 1:** The major diagnostic categories of the recruited cases.

	Total (n = 109)	Group 1 (Proteinuria <3 gm) (n = 47)	Group 2 (Proteinuria ≥3 gm) (n = 62)
**Immune-complex mediated glomerular diseases**	**31 (29%)**	**17 (36%)**	**14 (23%)**
Membranous GN	6	0	6
Lupus nephritis (Class II and Class V)	5	3	2
IgA Nephropathy (without crescents)	20	14	6
**Non-immune-complex mediated glomerular diseases**	**57 (52%)**	**14 (30%)**	**43 (69%)**
Diabetic glomerulosclerosis	31	4	25
Minimal change disease	8	1	7
Focal segmental glomerular sclerosis (FSGS)	11	7	4
Thin membrane nephropathy	3	1	2
Amyloidosis	1	0	1
Fibrillary GN	1	1	0
Myeloma cast nephropathy	1	0	1
Dense deposit disease	1	0	1
**Other kidney diseases**	**21 (19%)**	**16 (34%)**	**5 (8%)**
Mild to moderate chronic kidney injury	12	10	2
Acute tubular necrosis	7	5	2
Acute interstitial nephritis	2	1	1

Chi-square = 19.1, *p* < 0.001. The recruited cases were subdivided into immune-complex-mediated glomerular diseases, non-immune-complex-mediated glomerular diseases and other kidney diseases (as bolded in the table).

Demographic and clinical data of the patients are presented in **Table 2**. Patients in both groups were similar with regard to age (group 1: 48.5 ± 17 y.o. vs. group 2: 50.7 ± 17.2 y.o.; *p* = 0.4945), Ethnicity (*p* = 0.777), and sex (*p* = 0.815). Group 2 patients had higher systolic blood pressure than group 1 patients (144.2 ± 17.8 mmHg vs. 133.3 ± 18.8 mmHg, respectively; *p* = 0.0090).

**Table 2:** Patient demographic and clinical data.

		Total (n = 109)	Group 1 (Proteinuria <3 gm) (n = 47)	Group 2 (Proteinuria ≥3 gm) (n = 62)	p-values
Race	White	65 (72%)	27 (73%)	38 (72%)	0.777
	African American	17 (19%)	6 (16%)	11 (21%)	
	Others	8 (9%)	4 (11%)	4 (7%)	
Gender	M	64 (59%)	27 (57%)	37 (60%)	0.815
	F	45 (41%)	20 (43%)	25 (40%)	
Age (years)		50.1±16.9	48.5±17	50.7±17.2	0.4945
BP (mm Hg)	Systolic	139.3±18.9	133.3±18.8	144.2±17.8	0.0090
	Diastolic	81.6±14.0	80.2±11.5	82.7±15.7	0.4328
Serum creatinine, mg/dl		2.6±3.5	2.4±4.9	2.7±1.9	0.6484
BMI (kg/m^2^)		31.8±8.1	29.8±7.5	32.6±8.9	0.1435

M, males; F, females; BP, blood pressure; BMI, body mass index.

### Correlations between proteinuria and morphologic findings

**Figure 2:**
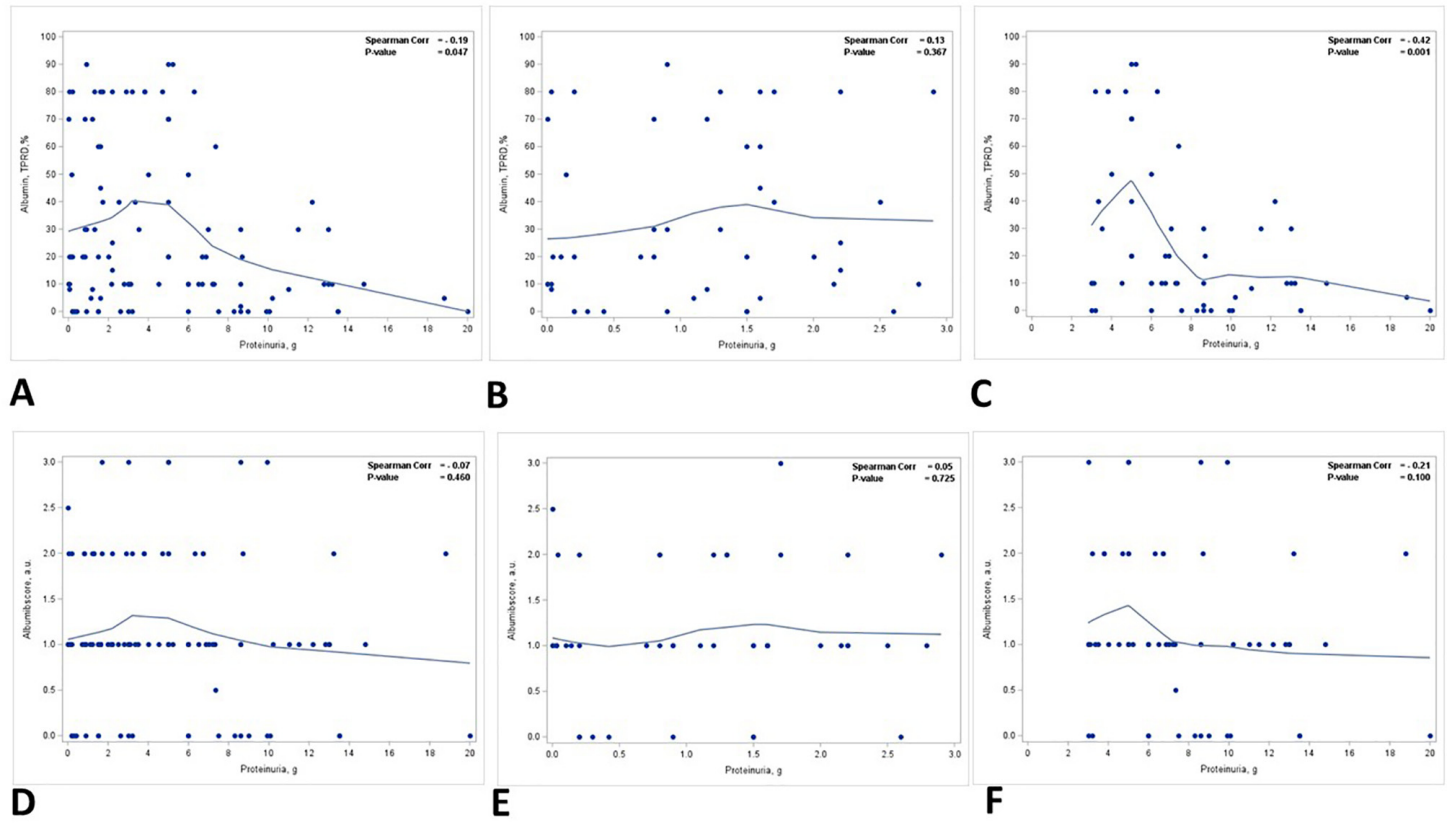
Correlation between proteinuria and immunofluorescence staining for albumin in TPRDs. Correlation between the percentage of tubules positive for albumin in TPRDs and proteinuria (g) in **(A)** all patients, **(B)** patients with proteinuria <3 g, and **(C)** patients with proteinuria ≥3 (g) Correlation between the staining intensity for albumin (graded from 0 to 3) in TPRDs and proteinuria (g) in **(D)** all patients, **(E)** patients with proteinuria <3 g, and **(F)** patients with proteinuria ≥3 (g) Spearman coefficient *R* and the corresponding *p*-value are shown on each plot with a fit line.

There was a reverse correlation between the staining for albumin in TPRDs and proteinuria. The correlation reached statistical significance only for the percentage of tubules with albumin TPRDs (*p* = 0.047) (**Figure 2A**) but not for the scoring intensity (*p* = 0.460) (**Figure 2D**). Subgroup analysis demonstrated that such correlation was stronger in group 2 patients with nephrotic range proteinuria, and similarly, only the correlation for the percentage of tubules with albumin reabsorption droplets reached statistical significance (*p* = 0.001) (**Figures 2C, F**). Conversely, such correlation was lost in group 1 patients with subnephrotic range proteinuria (**Figures 2B, E**).

**Figure 3:**
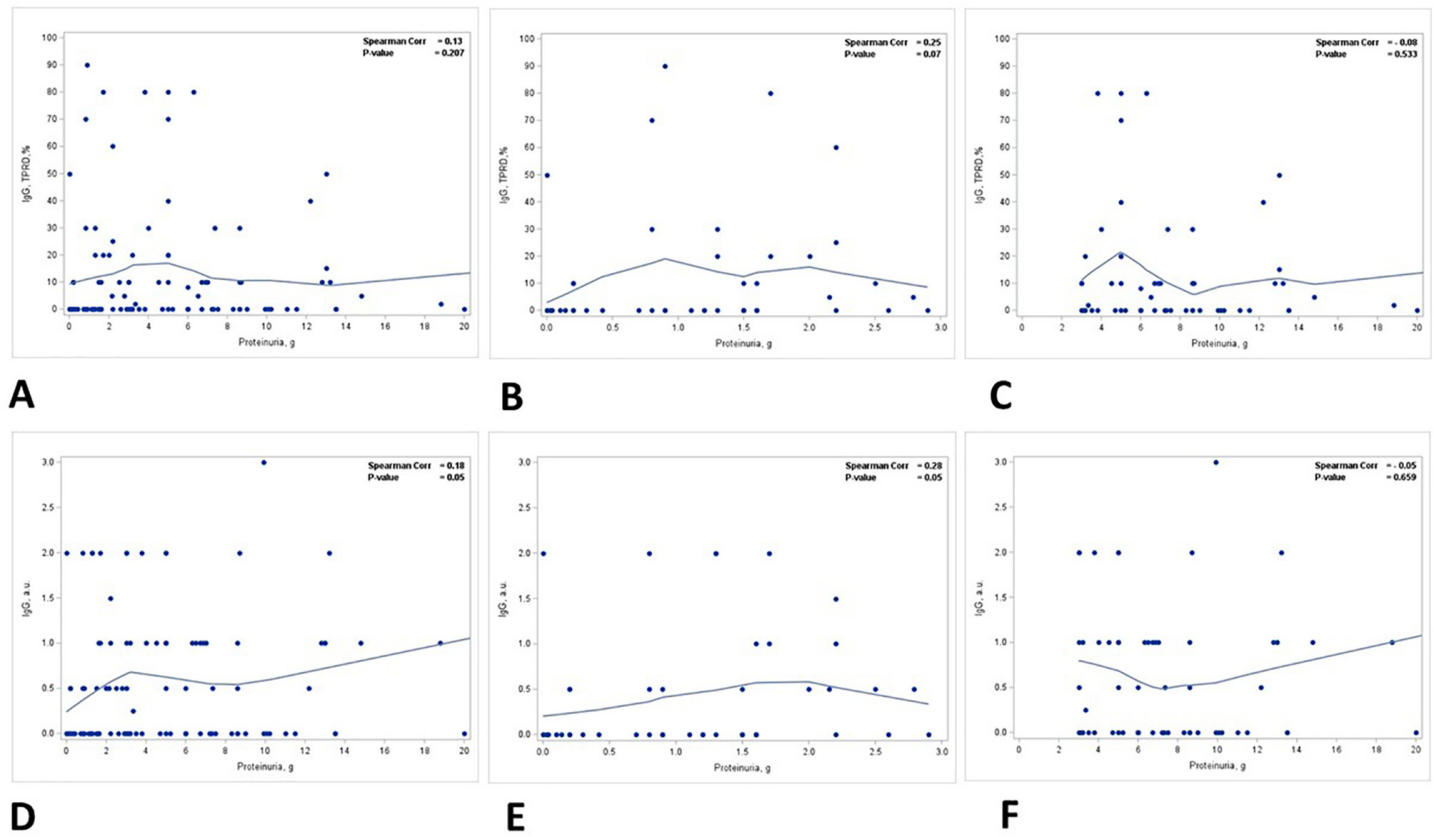
Correlation between proteinuria and immunofluorescence staining for IgG in TPRDs. Correlation between the percentage of tubules positive for IgG in TPRDs and proteinuria (g) in **(A)** all patients, **(B)** patients with proteinuria <3 g, and **(C)** patients with proteinuria ≥3 (g) Correlation between the staining intensity for IgG (graded from 0 to 3) in TPRDs and proteinuria (g) in **(D)** all patients, **(E)** patients with proteinuria <3 g, and **(F)** patients with proteinuria ≥3 (g) Spearman coefficient *R* and the corresponding *p*-value are shown on each plot with a fit line.

A positive correlation was identified between the staining intensity for IgG in TPRDs and proteinuria, but not the percentage of IgG TPRDs. In all patients, the correlation between the staining intensity for IgG TPRDs and proteinuria was on the verge of reaching statistical significance (*p* = 0.05) (**Figure 3D**) but not for the percentage of tubules with IgG TPRDs (*p* = 0.207) (**Figure 3A**). Similar correlations were observed in group 1 patients with subnephrotic range proteinuria (*p* = 0.05 for both the intensity and the percentage of IgG TPRDs staining, **Figures 3B, E**). However, this correlation was lost in group 2 patients with nephrotic range proteinuria (**Figures 3C, F**).

There were positive correlations between the percentage of globally sclerosed glomeruli, IFTA, vascular injury, podocyte foot process effacement, and proteinuria. The correlations were strongest for IFTA (%) and the degree of podocyte foot process effacement (**Table 3**).

**Table 3:** Relationships between histologic parameters and proteinuria.

Histologic parameter	Spearman correlation coefficient, *R*	*p*-value
Percentage of globally sclerosed glomeruli	0.2362	0.0143
Percentage of segmentally sclerosed glomeruli	−0.012	0.9062
IFTA (%)	0.391	<0.0001
Arterial intimal thickening	0.1272	0.1874
Arteriolar hyalinosis	0.3263	0.0005
Podocyte foot process effacement	0.5296	<0.0001

**Figure 4:**
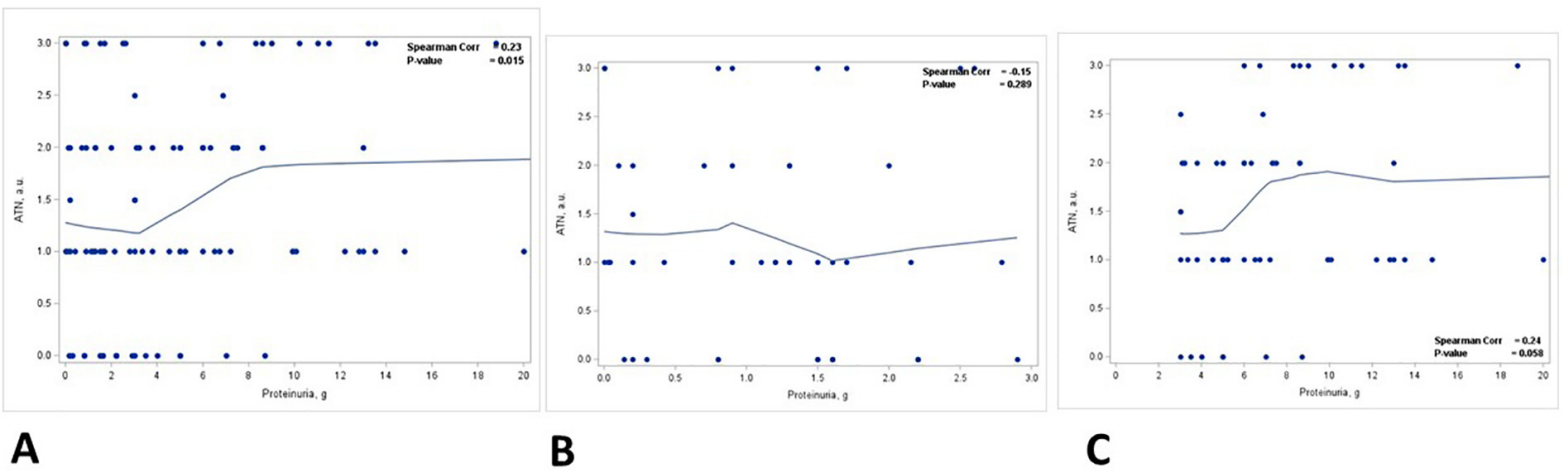
Correlation between proteinuria and acute tubular necrosis in kidney biopsies. Correlation between acute tubular necrosis (ATN) (graded from 0 to 3) and proteinuria (g) in **(A)** all patients, **(B)** patients with proteinuria <3 g, and **(C)** patients with proteinuria ≥3 (g) Spearman coefficient *R* and the corresponding *p*-value are shown on each plot with a fit line.

**Figure 5:**
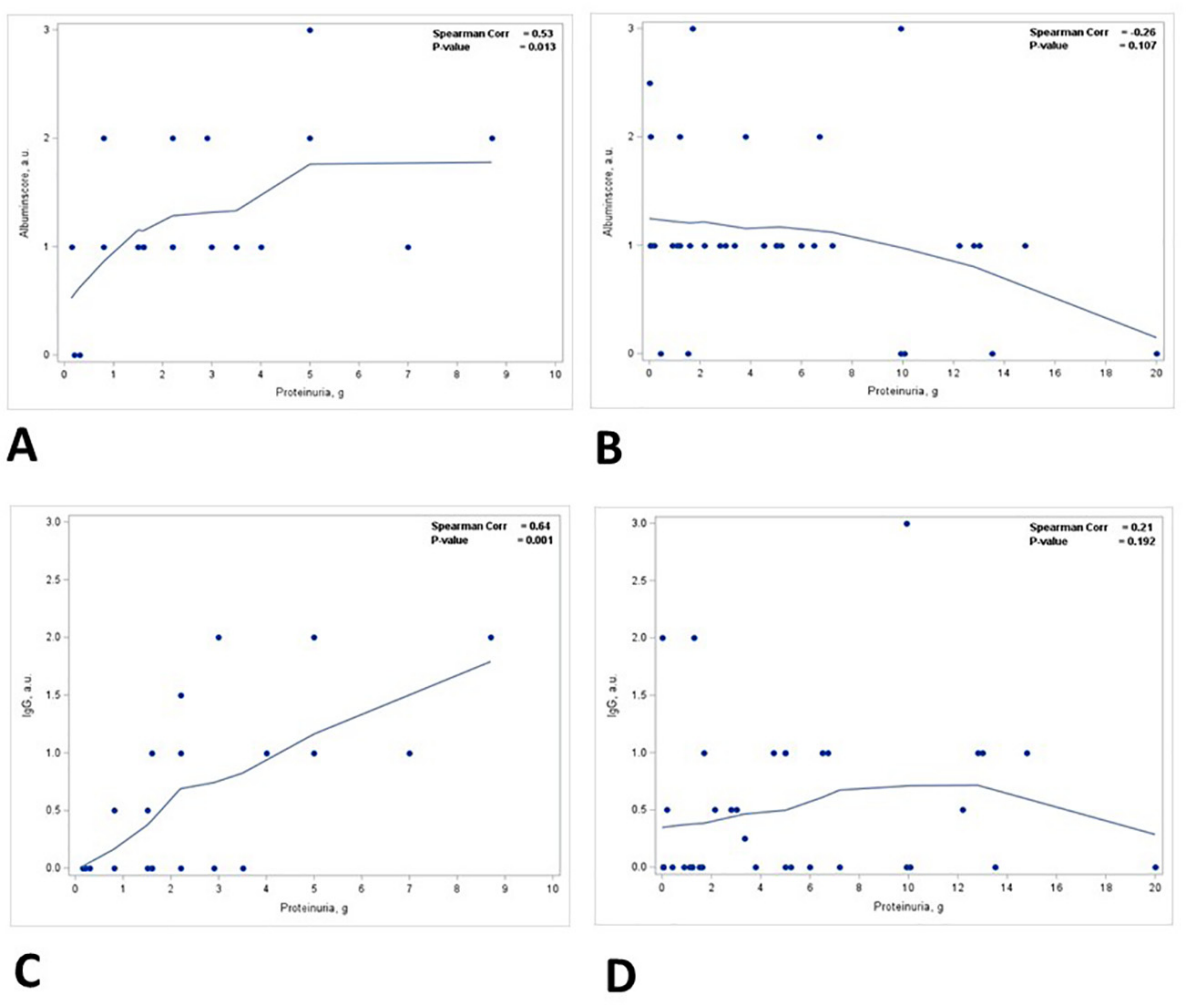
Correlation between proteinuria and staining intensity in TPRDs for albumin and IgG stratified based on the degree of ATN. Correlation between the level of proteinuria and the staining intensity in TPRDs for albumin in **(A)** cases with uninjured tubular epithelial cells (ATN = 0) and **(B)** in cases with injured tubular epithelial cells (ATN ≥ 1). Correlation between the level of proteinuria and the staining intensity in TPRDs for IgG in **(C)** cases with uninjured tubular epithelial cells (ATN = 0) and **(D)** in cases with injured tubular epithelial cells (ATN ≥ 1). Spearman coefficient *R* and the corresponding *p*-value are shown on each plot with a fit line.

There was a statistically significant positive correlation between the degree of ATN and the level of proteinuria (*p* = 0.015) (**Figure 4A**, **Table 3**). This correlation was noted in patients with nephrotic range proteinuria (group 2) (*p* = 0.058) (**Figure 4C**) but was lost in patients with subnephrotic range proteinuria (group 1) (**Figure 4B**). When the biopsies were stratified based on the presence or absence of ATN, there was a positive correlation between the level of proteinuria and the staining intensity in TPRDs for both albumin and IgG in cases with uninjured tubular epithelial cells (ATN = 0) (**Figures 5A, C**). This correlation was lost when tubular epithelial cells were injured (ATN ≥ 1) (**Figures 5B, D**, **Table 4**).

**Table 4:** Relationship between proteinuria and histologic parameters in patients with non-nephrotic (group 1) and nephrotic range (group 2) proteinuria.

Variable	Level	Group 1 (proteinuria < 3 g) (*n* = 47)	Group 2 (proteinuria >= 3 g) (*n* = 62)	Total (*n* = 109)	*p*-value
Albumin score	Median [IQR] (min, max)	1 [1, 2] (0, 3)	1 [1, 2] (0, 3)	1 [1, 2] (0, 3)	0.904
Albumin percentage	Median [IQR] (min, max)	20 [8, 60] (0, 90)	10 [5, 40] (0, 90)	20 [8, 50] (0, 90)	0.264
IgG score	Median [IQR] (min, max)	0 [0, 0.5] (0, 2)	0.5 [0, 1] (0, 3)	0 [0, 1] (0, 3)	0.051
IgG percentage	Median [IQR] (min, max)	0 [0, 10] (0, 90)	3.5 [0, 15] (0, 80)	0 [0, 10] (0, 90)	0.244
ATN	Median [IQR] (min, max)	1 [0, 2] (0, 3)	2 [1, 2] (0, 3)	1 [1, 2] (0, 3)	0.025
Percentage of globally sclerosed glomeruli	Median [IQR] (min, max)	15.4 [5.26, 36.36] (0, 85.71)	30 [6.25, 46] (0, 100)	23.08 [5.26, 40] (0, 100)	0.081
Percentage of segmentally sclerosed glomeruli	Median [IQR] (min, max)	0 [0, 5.56] (0, 50)	0 [0, 0] (0, 50)	0 [0, 0] (0, 50)	0.226
IFTA (%)	Median [IQR] (min, max)	10 [8, 22] (0, 65)	26 [12, 60] (0, 77)	20 [8, 35] (0, 77)	0.001
Arterial intimal thickening	Median [IQR] (min, max)	1 [0, 2] (0, 3)	1 [0, 2] (0, 3)	1 [0, 2] (0, 3)	0.290
Arteriolar hyalinosis	Median [IQR] (min, max)	1 [0, 1] (0, 3)	1.25 [0, 3] (0, 3)	1 [0, 2] (0, 3)	0.007
Podocyte foot process effacement	Median [IQR] (min, max)	20 [10, 50] (0, 100)	70 [50, 80] (10, 100)	50 [25, 80] (0, 100)	<0.0001

ATN, acute tubular necrosis; IFTA, interstitial fibrosis and tubular atrophy; IQR, interquartile range.

## Discussion

To the best of our knowledge, this is the first large-scale study to analyze the correlation between proteinuria and the specific histologic marker (TPRDs in PTECs) based on human kidney biopsies. Our data demonstrate that the extent of TPRDs negatively correlates with the level of proteinuria detected clinically. This is in concordance with the findings of the previous animal studies performed by Russo et al. (9) and Molitoris et al. (7), which have shown that PTECs possess the capacity to regulate albumin uptake and dysfunction of the albumin uptake pathway(s) in PTECs contributes to an increase in urinary albumin. Arafam et al. (10) studied the correlation between morphologically recognizable glomerular podocytes and renal tubular epithelium with reabsorbed proteins and the level of proteinuria in patients with various glomerulopathies. In that study, a total of 22 patients with clinical proteinuria and 3 patients as control were recruited. Similar to our data, the authors showed a negative correlation between the percentage of tubules with albumin protein reabsorption droplets and the level of proteinuria. We further analyze this finding by subdividing patients based on the proteinuria level. Subgroup analysis reveals that such correlation is stronger in group 2 patients with nephrotic range proteinuria.

Our study includes not only albumin tubular reabsorption droplets but also IgG tubular reabsorption droplets, analyzing selective and non-selective proteinuria. There is no clear relationship between IgG staining and proteinuria (**Figure 3**) until stratified by ATN. The relationship between semiquantitative IgG staining and proteinuria was borderline at *p* = 0.05. Both the scoring intensity and the percentage of tubules with IgG reabsorption droplets are higher in group 2 patients with nephrotic range proteinuria. IgG is an HMWP with a molecular weight of 150 kDa (11). Under normal physiological conditions, IgG accounts for only a small fraction of urinary proteins (5%–10%) (12) and the presence of a significant amount of urinary IgG signifies non-selective proteinuria (5). The extent of IgG reabsorption droplets in PTECs reflects the severity of proteinuria, and this can be a potential biomarker for non-selective proteinuria.

Our data also show the correlation between the degree of ATN and the level of proteinuria. The degree of ATN is more severe in group 2 patients with nephrotic range proteinuria (*p* = 0.025). A positive correlation is found between the degree of ATN and the level of proteinuria, but this correlation is present only in group 2 patients with nephrotic range proteinuria. When the biopsies are stratified based on the degree of ATN, there is a positive correlation between the level of proteinuria and the staining intensity in TPRDs for both albumin and IgG in cases without ATN. However, this correlation is lost when tubular epithelial cells are injured (i.e., cases with ATN ≥ 1). These data are supported by previous observations in animals that PTECs regulate the level of urinary albumin by changing its reabsorption (7). In previous animal studies, this was demonstrated by albumin only. Our data demonstrate that similar regulatory mechanisms are present for HMWP as well.

Persistent excessive protein exposure becomes toxic to PTECs by suppressing autophagy, which is an important cellular survival mechanism. Autophagy functions by constantly recycling macromolecules non-selectively to maintain cellular homeostasis, or by degrading certain targeted cellular components selectively to maintain cellular function under oxidative stress or starvation (8, 13). Chronic proteinuria inhibits autophagy in PTECs, resulting in an accumulation of toxic intracellular molecules including damaged mitochondria, thus leading to progressive tubular injury and demise of PTECs (8), which can manifest as IFTA by histology. On the other hand, dysfunction of PTECs in acute kidney injury (AKI) that histologically manifests as ATN (14) can also lead to proteinuria. In either condition, the damaged PTECs lose their ability to reabsorb proteins that are filtered through the GFB, further aggravating the degree of proteinuria and potentially resulting in a vicious cycle. This explains why a positive correlation between the degree of proteinuria and the amount of reabsorbed proteins in the tubules is present in cases without ATN, but this correlation is lost whenever there is any injury to the tubular epithelial cells. Studies performed on rats with doxorubicin-induced nephrosis or age-related proteinuria confirmed that the accumulation of filtered proteins in the PTECs was associated with IFTA and glomerulosclerotic lesions (15, 16). More recent studies have discovered that chronic proteinuria substantially increases the synthesis of reactive oxygen species (ROS) in PTECs, which triggers and exacerbates tubulointerstitial inflammation and fibrosis (8).

Our findings of positive correlations between the percentage of globally sclerosed glomeruli as well as IFTA and the level of proteinuria are in agreement with the results of animal studies. In addition, the percentage of globally sclerosed glomeruli and IFTA is higher in group 2 patients with nephrotic range proteinuria. All these imply that the level of proteinuria depends on the functional renal cortex. This is probably related to the progressive nephron loss with subsequent worsening of glomerular hyperperfusion/hyperfiltration injury at the single nephron level.

As expected, there is a strong positive correlation between the degree of podocyte foot process effacement and the level of proteinuria, and the degree of foot process effacement is more severe among patients with nephrotic range proteinuria (*p* < 0.0001). The exact significance of the degree of arteriolar hyalinosis in kidney biopsies is yet to be determined. Studies have shown that arteriolar hyalinosis is related to rapid GFR decline (17) and predicts the onset of both macroalbuminuria and eGFR <60 in patients with type 2 diabetes (18). Our data demonstrate a strongly significant positive correlation between the degree of arteriolar hyalinosis and the level of proteinuria (*p* = 0.0005). Although the precise mechanism underlying this correlation remains unclear, it is possible that hyaline deposition reduces the lumen and elasticity of arterioles. This could interfere with the glomerular filtration pressure and post-glomerular oxygen supply of the tubules, worsening the level of proteinuria both at the levels of glomerular filtration and tubular protein reabsorption.

There are several limitations in our studies. First, for most of the correlations, significance is near *p* = 0.05 with a low Spearman correlation coefficient (0.1–0.2). These identify weak correlations that could be related to selection criteria or a low number of patients. On the other side, there are strong correlations after the correction for ATN (Spearman > 0.5 and *p* < 0.001). Second, there are several ways to distinguish selective from non-selective proteinuria (albuminuria vs. immunoglobulinuria). In our studies, we used only general proteinuria because of the limited data regarding the specific composition of the proteins in the urine which is usually not a part of a routine clinical workup. Our results would have been stronger if the correlations had taken into account not only proteinuria but also albuminuria or urinary B2 microglobulin. Third, as it was mentioned above, statistical power is somewhat low, which could be due to possible inclusion bias and sample size. Fourth, due to a lack of data on urinary sediment analysis in many patients, it was not included in the analysis. Urine sediment data could help in determining the degree of ATN. Fifth, the semiquantitative scoring of IgG or albumin uptake (1+ to 3+, etc.) may be unreliable due to subjectivity and the focal nature of proteinuric renal diseases. These data seem to suggest that reporting it as a percentage of proximal tubular cells with TRPDs is a more robust measurement of these underlying mechanisms.

Even though the clinical significance of our findings at this moment is not clear, these data indicate the similarity between the experimental animal studies and human biopsy findings, suggesting that similar mechanisms of renal function regulation are present. Therefore, pharmacological and therapeutic studies on animals are valid for humans as well.

## Conclusions

Our data support the hypothesis that PTECs have the ability to regulate protein reabsorption, thus regulating proteinuria. This function depends on the functional integrity of PTECs and such correlation is lost in cases with ATN. Also, the level of proteinuria correlates with the degree of podocyte foot process effacement and the degree of chronic kidney injury.

## Data Availability

The raw data supporting the conclusions of this article will be made available by the authors, without undue reservation.
